# A generic HTS assay for kinase screening: Validation for the isolation of an engineered malate kinase

**DOI:** 10.1371/journal.pone.0193036

**Published:** 2018-02-20

**Authors:** Romain Irague, Christopher M. Topham, Nelly Martineau, Audrey Baylac, Clément Auriol, Thomas Walther, Jean-Marie François, Isabelle André, Magali Remaud-Siméon

**Affiliations:** 1 Laboratoire d’Ingénierie des Systèmes Biologiques et Procédés, LISBP, Université de Toulouse, CNRS, INRA, INSA, Toulouse, France; 2 Toulouse White Biotechnology, Parc technologique du canal, Bâtiment NAPA CENTER B, Toulouse, France; Universite Paris-Sud, FRANCE

## Abstract

An end-point ADP/NAD^+^ acid/alkali assay procedure, directly applicable to library screening of any type of ATP-utilising/ADP producing enzyme activity, was implemented. Typically, ADP production is coupled to NAD^+^ co-enzyme formation by the conventional addition of pyruvate kinase and lactate dehydrogenase. Transformation of enzymatically generated NAD^+^ into a photometrically active alkali derivative product is then achieved through the successive application of acidic/alkali treatment steps. The assay was successfully miniaturized to search for malate kinase activity in a structurally-guided library of LysC aspartate kinase variants comprising 6,700 clones. The screening procedure enabled the isolation of nine positive variants showing novel kinase activity on (L)-malate, the best mutant, LysC V115A:E119S:E434V exhibited strong substrate selectivity for (L)-malate compared to (L)-aspartate with a (*k*_cat_/*K*_m_)_malate_/(*k*_cat_/*K*_m_)_aspartate_ ratio of 86. Double mutants V115A:E119S, V115A:E119C and E119S:E434V were constructed to further probe the origins of stabilising substrate binding energy gains for (L)-malate due to mutation. The introduction of less sterically hindering side-chains in engineered enzymes carrying E119S and V115A mutations increases the effective volume available for substrate binding in the catalytic pocket. Improved binding of the (L)-malate substrate may be assisted by less hindered movement of the Phe184 aromatic side-chain. Additional favourable long-range electostatic effects on binding arising from the E434V surface mutation are conditionally dependent upon the presence of the V115A mutation close to Phe184 in the active-site.

## Introduction

Kinases are essential enzymes involved in a wide variety of metabolic and regulatory functions in all biological systems. Although they are predominantly intracellular enzymes, extracellular kinases have been recently identified.[1–4] These enzymes catalyse phosphoryl transfer reactions from ATP to an acceptor molecule which can be small molecules such as monosaccharides, organic acids, nucleotides and amino acids as well as larger ones including lipids and proteins. The prevalence of this reaction in nature, has made kinases an important target enzyme class in drug discovery.[5] Moreover, engineering or discovery of new kinases are likely to increase with advances in synthetic biology as these enzymes catalyse reactions that can be thermodynamically critical for the successful *in vivo* implementation of synthetic pathways.

Kinase engineering was one of the challenges that we encountered in the implementation of a synthetic metabolic pathway in *Escherichia coli* for the production of the non-natural metabolite 2,4-dihydroxybutyric acid (DHB) that can serve as a precursor molecule for the chemical synthesis of several added-value (bio)chemicals including methionine.[6] Indeed, DHB is not produced by living cells, but the sequential action of malate kinase, malate semialdehyde dehydrogenase and malate semialdehyde reductase was envisaged as a feasible enzyme cascade for the three-step conversion of (L)-malate into DHB.[6] Malate kinase (MK) activity has not been previously described in living organisms, and therefore, a malate kinase enzyme had to be engineered. As malate differs from aspartate only by the presence of a hydroxyl group in place of an amino group at the C2 (α) carbon, an aspartate kinase from *E*. *coli* was used as template for enzyme design. Three different Aspartate kinase (AK) forms exist in *E*. *coli*, two of which are bifunctional enzymes, possessing an additional homoserine dehydrogenase activity.[7] To facilitate enzyme engineering, the mono-functional AKIII[8], encoded by *lysC*, was the preferred template enzyme. Although the enzyme has a broad substrate specificity, (L)-aspartate analogues comprising a substituted α-amino group are not substrates, but are instead weak competitive inhibitors (*K*_i_, malate = 53 mM).[9] It was thus necessary to re-design the LysC enzyme in order to accommodate (L)-malate as a substrate.

We have employed a structurally-guided approach to identify relevant amino acid residues as target sites for mutagenesis in order to convert this aspartate kinase into a catalytically effective malate kinase, and set up a rapid and effective screening assay enabling the isolation of active variants with the desired enzyme activity. An efficient end-point microplate assay was devised and applied to screen a LysC mutant library of approximately 6,700 clones. The protocol, adapted from the alkalization-dependent NAD(H) alkali assay originally proposed by Tsotsou et al.[10] allowed the identification of 9 positive mutants that were individually confirmed to be active. The molecular origins of the substrate specificity have been investigated by step-wise reconstruction of the best malate kinase mutant. The results demonstrate the wide utility of the generic screening assay in the isolation of kinase mutants of interest from large libraries and in the identification of new kinase substrates or inhibitors.[11]

## Materials and methods

### Materials

Unless otherwise specified, all materials were purchased from Sigma-Aldrich (St-Louis, MO).

### Bacterial strains, plasmids and growth conditions

NEB 5-α Competent *E*. *coli* (New England Biolabs) was used for mutant library construction. One shot^®^*E*. *coli* BL21 Star^™^ (DE3) (Invitrogen) was used to set-up the screening method for library screening and the larger scale production of selected variants. Plasmid pET28 harbouring the gene LysC in frame with a sequence coding a N-terminal 6xHis tag was used as template in the construction of the LysC mutant library. Bacterial strains were grown on LB medium supplemented with 50 μg U.mL^-1^ kanamycin at 37°C.

### DNA manipulations

Restriction endonucleases and DNA-modifying enzymes were purchased from New England Biolabs and used according to the manufacturer’s instructions. DNA plasmid isolation was performed using the GeneJET Plasmid Miniprep Kit (Thermo scientific). DNA extraction from agarose gel was carried out using the GeneJET Gel Extraction Kit (Thermo scientific). DNA sequencing was carried out by Beckman Coulter Genomics (Takeley, UK).

### Organisation and storage of *E*. *coli* clones

Colonies of *E*. *coli* clones generated after transformation were picked from solid LB medium and transferred onto 96-well microplates containing 200 μL of LB medium supplemented with 8% glycerol in each well. Transfer was carried out using a QpixII colony picker (Genetix, Hampshire, UK). Microplates were stored at -80 °C after overnight culture at 30°C.

### Growth and recombinant protein expression conditions in 96-deepwell format

Storage microplates were thawed and replicated to inoculate starter culture into 96-well microplates (Nunc^™^ Brands Products, Roskilde, Denmark) filled with 200 μL 2xYT medium per well supplemented with kanamycin (50 μg.mL^-1^). After 24 h growth at 30°C under horizontal shaking conditions at 250 rpm, 50 μL aliquots of each starter culture were used to inoculate 96-deep well plates (ABgene, Epsom, UK) containing 1 mL of auto-inducing media ZYM-5052[12] supplemented with kanamycin (50 μg.mL^-1^). Growth and gene expression were conducted for 24 h at 30°C, 700 rpm, in an incubator-shaker (INFORS HT, Bottmingen, Switzerland).

### ADP/NAD^+^ acid/alkali assay set up

The experimental protocol was inspired by a previously reported procedure for the detection of NADP^+^ co-enzyme generated by oxidoreductase activity.[10,11] The method is based on the addition of a strong acid (0.3 M HCl) to stop the reaction and to degrade any unreacted NADPH, leaving any NADP^+^ co-enzyme present unchanged. Strong alkali (9.0 M NaOH) is then added to generate a derivative of NADP^+^ that displays an absorption maximum at 360 nm.[14,15] This assay was adapted to NAD^+^ formation.

#### Effect of sequential acid/alkali treatment on NADH or NAD^+^co-enzymes

Stock solutions of 0.5 mM NADH or NAD^+^ were diluted 1:2 v/v with 0.3 M HCl and incubated at room temperature for 20 to 25 min. The acid treated solutions were then diluted again at 1:4.2 v/v ratio with 9 M NaOH. The reaction solution was incubated at room temperature in the dark for 3 h. UV-Vis spectra were recorded at each stage on 250 μL aliquots of pure, acid-treated and alkali treated solutions using a BioTek^™^ Eon^™^ Microplate Spectrophotometer. In the evaluation of the variation of absorbance as a function of the NAD^+^ alkali derivative concentration, NAD^+^ solutions in the concentration range from zero to 12.5 mM were diluted at 1:2 v/v ratio with 0.3 M HCl, incubated at room temperature for 20 to 25 min and then diluted further at 1:4.2 v/v ratio with 9 M NaOH, as described above before UV absorbance monitoring at 360 nm. The sensitivity of the assay method was determined as the limit of detection (LOD) as follows:

LOD=3σ/b

where *σ* is the standard deviation of the response on the negative ordinate intercept and *b* is the slope of the absorbance curve at low NAD^+^ alkali derivative concentrations.

#### Enzyme activity determination in microplates using the ADP/NAD^+^ acid/alkali assay

After incubation, the culture plates were centrifuged (10 min, 3,700 g, 4°C) and the supernatants removed. Bacterial cell pellets were re-suspended in 200 μL of lysozyme solution (0.5 mg.mL^-1^), incubated 30 min at 30°C and frozen at -80°C for 12–24 h. After thawing for 1 h at room temperature, 800 μL of benzonase solution (2.4 U.mL^-1^) were added to each well and plates incubated for 30 min at 30 °C before centrifugation (10 min, 3,700 g, 4°C). Enzymatic extracts were diluted 50-fold with water. 10 μL of diluted enzymatic extracts were then transferred onto a new microplate together with 65 μL of reaction mixture (115 mM HEPES buffer pH 7.5, 60 mM KCl, 6 mM MgCl_2_, 5 mM ATP, 3.85 mM phosphoenolpyruvic acid, 2.88 mM NADH, 3.85 U.mL^-1^ lactate dehydrogenase and 3.85 U.mL^-1^ pyruvate kinase) and the reaction started by the addition of 50 μL of 50 mM neutralised substrate. Reactions were carried out for 40 min at 30 °C and then stopped by the addition of 125 μL of 0.3 M HCl. After 20 min incubation at room temperature, 60 μL of reaction media were transferred onto a new microplate containing 190 μL of 9 M NaOH (1:4.2 V/V dilution ratio). Microplates were left in the dark for 3 h at room temperature to allow the development of NAD^+^ alkali product derivatives, after which the absorbance at 360 nm was recorded using a BioTek^™^ Eon^™^ Microplate Spectrophotometer. The NAD^+^ concentration (and therefore concentration of the ADP product) obtained during 40 min incubation was determined from the calibration curve, and then used to calculate kinase activity in the samples.

#### Reproducibility and robustness of the screening procedure

The reproducibility of the screening process was evaluated by the determination of the coefficient of variation (CV) of aspartate kinase activity of recombinant LysC calculated as:

$$CV\;=\;100\;\sigma_{p\;}/\mu_{p}$$

where *σ*_*p*_ and *μ*_*p*_ are the standard deviation and mean of the measured aspartate kinase activities. The Z’ factor statistical metric of the robustness of the method was calculated according to the following equation:

$$Z\prime \;factor\;=\;1-(3\sigma_{p}+\;3\sigma_{n})/{\;|\mu }_{p}\;-\mu_{n}|$$

where *μ* and *σ* are the data mean and standard deviation respectively, “*p*” refers to the positive control and “n” to the negative control.

### Generation of LysC mutant library

Mutagenesis was carried out using the Incorporating Synthetic Oligonucleotides via Gene Reassembly (ISOR) method [16]. Briefly, primers pETseq_FOR and pETseq_REV were used to amplify the LysC gene from the plasmid pET28_LysC (S1 Table). The purified PCR product was digested with DNaseI endonuclease under conditions favouring fragment generation of average length 100 bp. Gel purified fragments (_~_ 100 ng) were used for gene reassembly in combination with 2 μM of degenerated oligonucleotides (S1 Table). The reaction mixture (30 μL), containing 1 U Phusion^®^ High-Fidelity DNA Polymerase (Finnzyme) in the appropriate buffer and 0.4 mM of each dNTPs, was thermocycled according to the following programme: 1 denaturation step at 98°C for 30 s; 40 cycles comprising a denaturation step at 98°C for 10 s, 6 successive 4°C hybridization steps each, from 65 to 41°C for 10 s each and an elongation step at 72°C for 20 s; and finally a 2 min step at 72°C. The fully recombined genes were isolated from the reassembly products by nested PCR using the primers Ec_lysC_clon_for and Ec_lysC_clon_rev primers. The PCR product was gel purified, digested with NdeI and BamHI and ligated into the corresponding sites of plasmid pET28. NEB 5-alpha competent *E*. *coli* strain was transformed with the ligation product and cultivated overnight at 37°C onto LB agar supplemented with kanamycin (50 μg/mL). Colonies were scraped from the plates for plasmid extraction and the DNA library obtained was used to transform chemo-competent *E*. *coli* BL21 Star^™^ (DE3) cells. A total of 6,720 generated colonies were picked and transferred into 96-well microplates containing 200 μL of LB medium supplemented with antibiotic and 8% glycerol in each well. The microplates were stored at -80 °C after overnight culture at 30°C.

### Production and purification of wild-type enzyme and mutants with altered specificities

*E*. *coli* cells carrying the pET28 plasmid encoding the 6xHis N-terminal tagged catalyst were cultivated in flask to an *A600* of approximately 0.6, at which point the cultures were induced with 1 mM IPTG. After a three hour period of induction, the cells were harvested by centrifugation (5432 *g*, 15 min, 4 °C). The pellet was re-suspended in wash buffer (50 mM HEPES, 30 mM NaCl, pH 7.5). After sonication, the extract was centrifuged (2057 *g*, 15 min, 4 °C) and the supernatant harvested for subsequent purification. 6xHis-tagged proteins were purified using TALON^®^ Metal Affinity Resin (Clontech) according to the manufacturer’s protocol. The protein content was determined by the micro Bradford method[17] using bovine serum albumin as standard.

### Enzyme assays

#### Aspartokinase III (LysC)

The enzyme activity was determined in a coupled assay by monitoring the oxidation of NADH at 340 nm, using a microplate spectrophotometer reader (Versamax, Molecular Devices) thermostated at 30°C. The coupled assay mixture contained 60 mM HEPES buffer (pH 7.5), 60 mM KCl, 6 mM MgCl_2_, 1 mM ATP (neutralised with NaOH), 0.25 mM NADH, 0.8 mM phosphoenolpyruvic acid (neutralised with NaOH), 2 U.mL^-1^ lactate dehydrogenase and 2 U.mL^-1^ pyruvate kinase. The enzyme reaction was initiated by the addition of (L)-aspartic acid at 50 mM. For kinetic parameter determination initial (L)-aspartic acid or ATP concentrations were varied from 0.1 to 50 mM and 0.08 to 20 mM, respectively. One activity unit is defined as the amount of enzyme producing 1 μmol of ADP per minute at 30°C.

#### Malate kinase (LysC mutants)

The enzyme activities were determined according to the procedure used for LysC aspartate kinase except that ATP was used at a concentration of 3 mM with (L)-malate as the substrate.

### Computational methods

Molecular models of V115A:E119S and V115A:E119S:E434V mutant aspartate kinase III (LysC) ternary complexes with (L)-malate and Mg-ADP were built and energy minimised as previously described [6]. Electrostatic interaction energy calculations on LysC mutant complexes with (L)-malate were performed using in house MFPB software. The program outputs Coulombic and reaction field contributions to the total electrostatic energy. The linear Poisson-Boltzmann equation was solved iteratively on a finite difference grid with 0.5 Å spacing using the Successive Over Relaxation (SOR) method.[18] Partial charges sets were the same as those used in energy minimisation. The Connolly molecular surface used to define the dielectric boundary was mapped onto the grid using a modified set of Bondi (1964) atomic radii[19, 20] and a solvent probe radius of 1.4 Å. Polar solvation free energy calculations were performed at 298K and 0.145M ionic strength. The protein interior dielectric constant was set to either 2 or 4, and the external dielectric constant for the continuum water solvent was 78.54.

## Results and discussion

### Screening assay set–up

**Fig 1:**
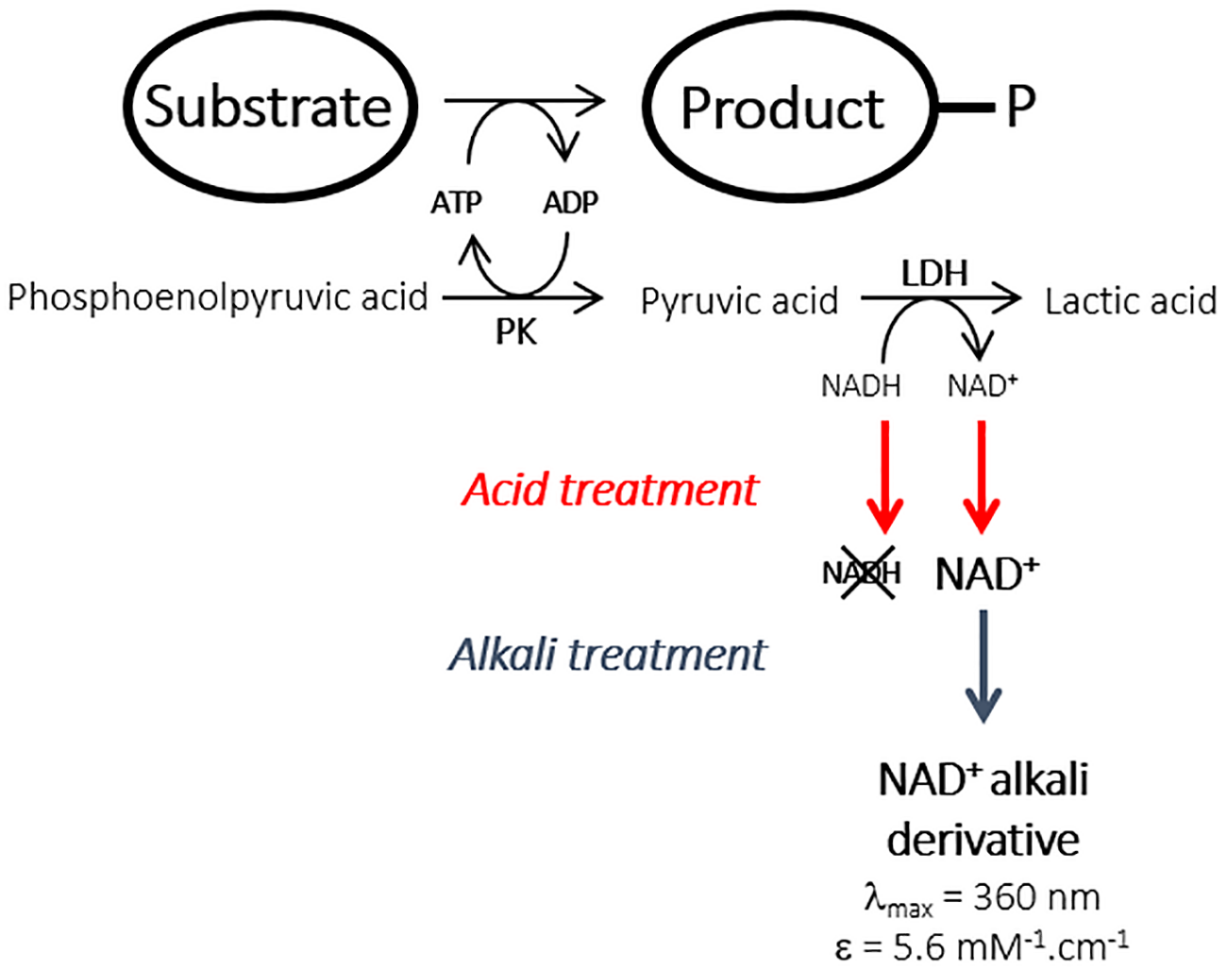
Principle of the end-point alkali assay method for the high-throughput screening of kinase activity.

The feasibility of an end-point ADP-based kinase assay was investigated with the prime objective of modifying kinase substrate specificity (Fig 1). Indeed, ATP-utilizing enzyme activity is often determined by the well-established ATP/NADH assay in which the ATP consumption rate is related to the rate of decrease in NADH concentration using a pyruvate kinase/lactate dehydrogenase coupled enzyme system.[21–23] The assay has been previously adapted to microtiter plate assays[21,23] but was found not to be well suited to high-throughput library screening, notably for the isolation of low activity variants. This prompted us to develop an ADP/NAD^+^ end-point assay based on the direct estimation of NAD^+^ production after a fixed reaction time.

**Fig 2:**
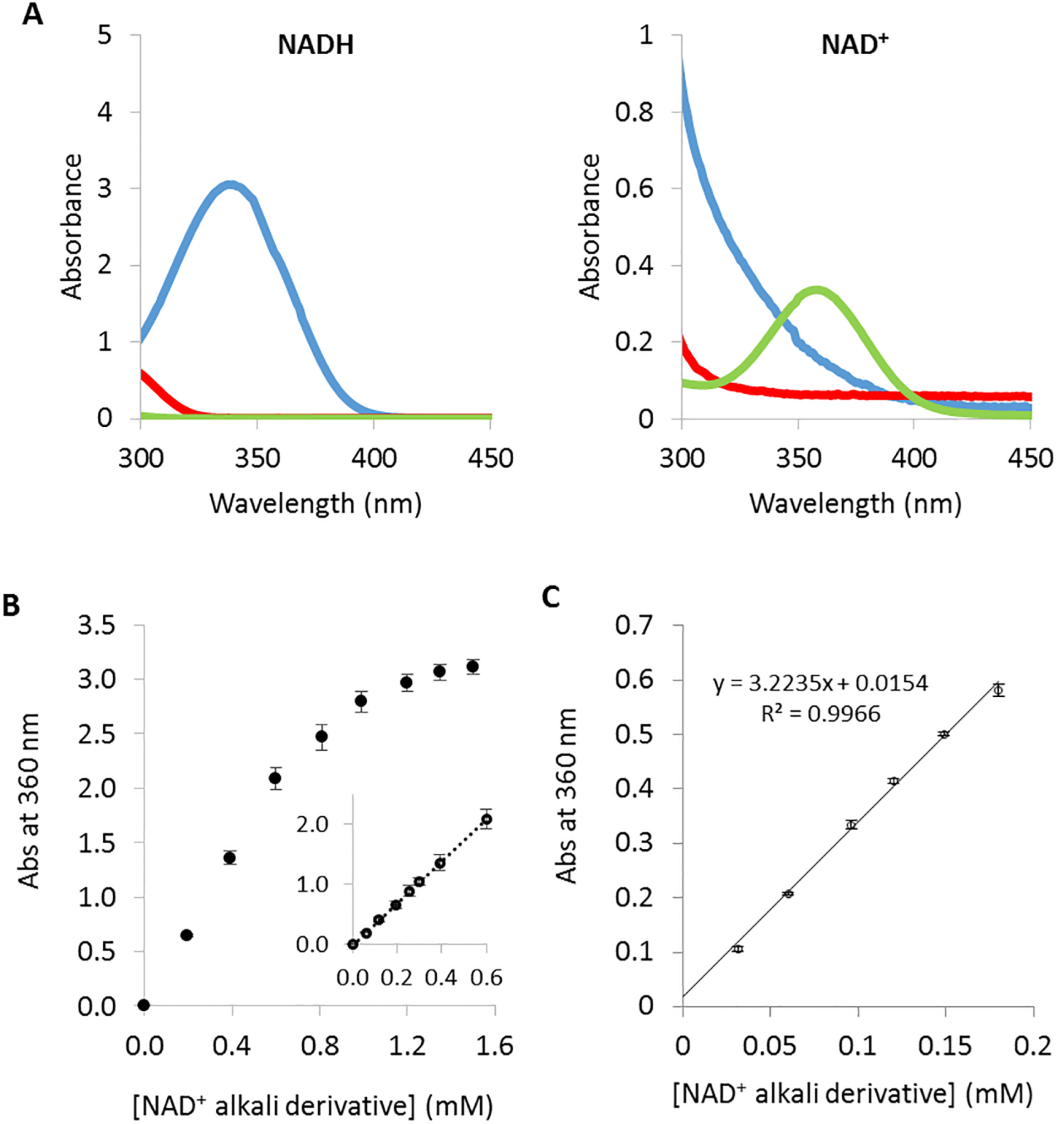
Screening assay set-up. (A) Absorbance spectra of NADH and NAD^+^ co-enzyme solutions, at an initial concentration of 0.5 mM, during the sequential acid/alkali treatment. UV-visible spectra were recorded on solutions of the pure co-enzyme forms (blue curves), after acid (red curves) and alkali treatment (green curves). (B) Absorbance at 360 nm, determined in microplate format, as a function of NAD^+^ alkali derivative concentration assuming the total conversion of the NAD^+^ co-enzyme initially present. (C) NAD^+^ alkali derivative calibration curve, determined in microplate format, using solutions mimicking a reaction medium, typically containing inactivated enzyme crude extract, aspartate, ATP and a varying NADH/NAD^+^ co-enzyme ratio at a fixed final total concentration of the reduced and oxidised forms of 1.5 mM.

The sequential acid/alkali treatment described in [13] was first applied to pure solutions of either 0.5 mM NADH or NAD^+^. Absorbance spectra were recorded at each step in the treatment (Fig 2A). The results showed that decreasing the pH to approximatively 1 led to the irreversible destruction of the reduced co-enzyme as revealed by the loss of absorbance signal at 340 nm. In contrast, the NAD^+^ UV-Vis spectrum was found to be unaffected taking into account the dilution due to addition of the acidic solution. Furthermore, the subsequent increase in the pH to 13 using NaOH led to the expected generation of the NAD^+^ alkali derivative that absorbed at 360 nm with a molar extinction coefficient of 5.6 mM^-1^.cm^-1^. As seen in Fig 2B, a linear response was obtained in the concentration range from zero to 0.6 mM of the NAD^+^ alkali derivative, corresponding to an initial NAD^+^ co-enzyme concentration range from zero to 5 mM when allowance is made for the dilution factor (1:8.4) due to the sequential acid/alkali treatment. The detection limit was estimated to be 6 μM NAD^+^ alkali derivative in microplate format. This detection limit corresponds to an initial NAD^+^ concentration of 50 μM formed in the reaction mixture, assuming the complete conversion of the NAD^+^ to alkali derivatives.

We then verified the linearity of NAD^+^ detection in microtiter plate format using an inactive surrogate reaction medium containing 10 μL of heat inactivated crude enzyme extract (typically consisting of a preparation of LysC with an activity of 0.4 U.mL^-1^ before thermal denaturation) and 115 μL of reaction medium containing variable ratios of NADH/NAD^+^ at a fixed final total concentration of the reduced and oxidised forms of 1.5 mM. The amount of inactivated crude enzyme extract used in this preliminary assay approximately corresponds to the amount of active enzyme needed to catalyse the formation of a maximum of 1.5 mM NAD^+^ according to the screening conditions described in the experimental section. As can be seen in Fig 2C, a linear correlation was obtained between absorbance and the increase of NAD^+^ concentration (itself related to ADP formation) showing that the assay could be applied to the screening of the LysC variant library. Similar results were obtained when aspartate was replaced by malate as the substrate.

### Validation on a library of LysC aspartate kinase variants

**Fig 3:**
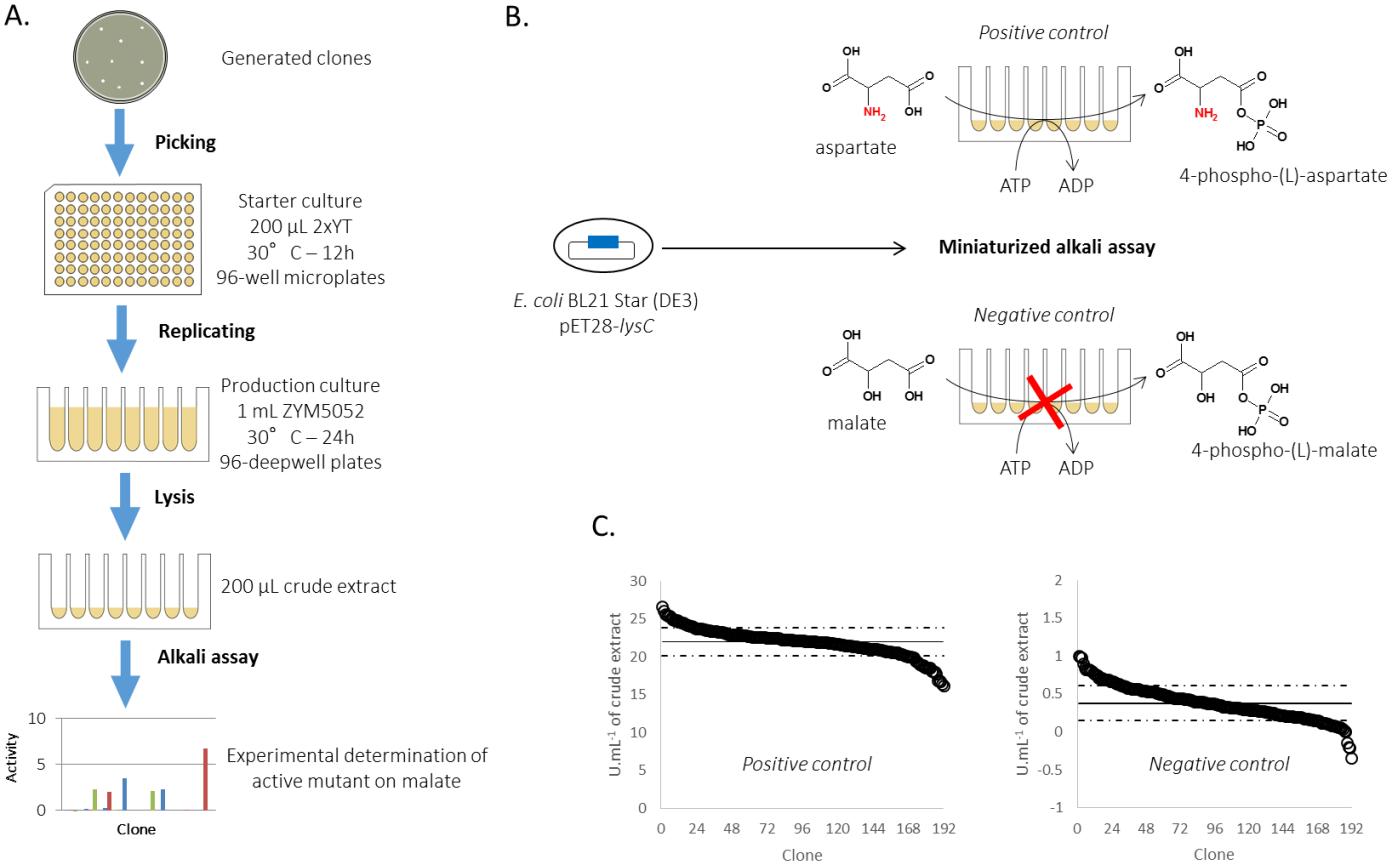
High-throughput method used to screen LysC mutants active on malate. (A) Procedure for the identification and isolation of recombinant malate kinase enzyme from generated clones. (B) Screening method validation strategy. (C) LysC activities on the natural substrate aspartate (positive control) and the non-natural substrate (L)-malate (negative control) determined for a single microplate. Activities (black circles) are plotted in descending order. Mean activities and their standard deviations are shown as solid and dotted horizontal black lines, respectively.

The general, miniaturized and fully automated procedure for the screening of a kinase mutant library is illustrated in Fig 3A. The method encompasses (i) the organisation of a library of clones expressing kinase mutants in microplate format, (ii) cell culture and recombinant enzyme expression, (iii) crude protein extract preparation and (iv) kinase activity determination. Validation of the entire procedure was carried out prior to the screening of larger kinase mutant libraries. Plasmid pET28-LysC was used to transform competent *E*. *coli* cells. From the clones obtained after transformation, 192 were picked from agar plate to measure aspartate kinase (positive control) and malate kinase (negative control) activities using the ADP/NAD^+^ acid/alkali assay as depicted in Fig 3B. Reactions were allowed to proceed for 40 min before acid/alkali treatment. An average aspartate kinase activity of 22± 2 U.mL^-1^ in the crude extract was obtained. The high reproducibility of the method is demonstrated by a correspondingly low coefficient of variation of 8.4%. When malate is used as the substrate, an overall average activity of 0.4 ± 0.2 U.mL^-1^ of crude extract was obtained. The latter result represents the background activity and accounts for less than 2% of the average aspartate kinase activity. This background can probably be accounted for by the presence of endogenous non-specific NADH-utilising oxidoreductase enzymes and possible transformation of the remaining aspartate present in the *E*. *coli* extract. A Z’ statistical factor (see Methods) of 0.71 was determined from these results, demonstrating the robustness of the method.

**Fig 4:**
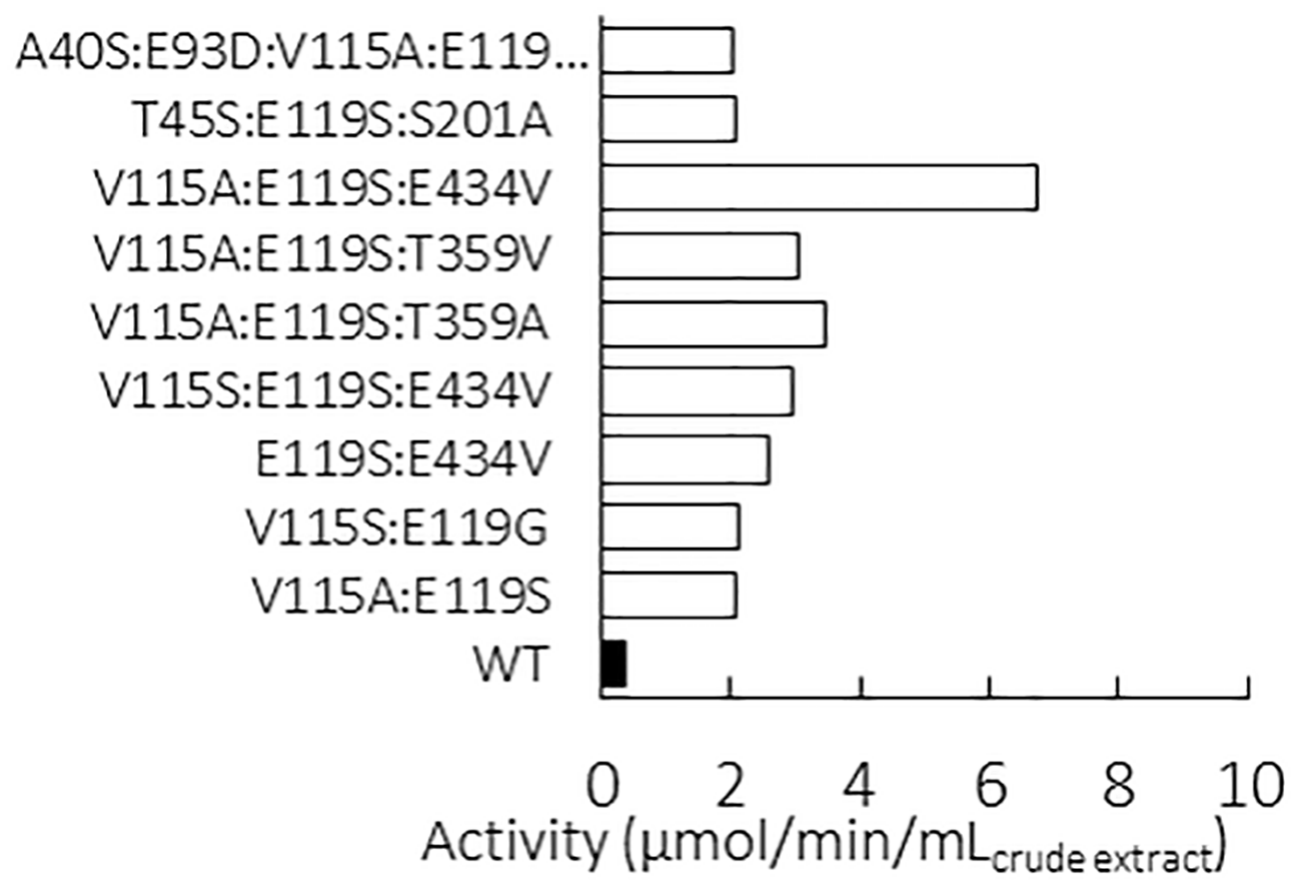
Malate kinase activities of the nine positive clones identified by screening of crude cell extracts. A control comparison is made with a clone expressing the wild-type enzyme (WT), corresponding to non-specific and/or malate-dependent endogenous NADH-dependent oxidoreductase enzymes.

The procedure was used to screen a structurally-based designed library of LysC mutants as previously described in [6]. Briefly, nine amino acid residue positions in the active site that could be targeted by mutagenesis to improve (L)-malate recognition were identified from comparative structural analyses of (L)-aspartate and (L)-malate binding interactions with LysC in experimental X-ray and 3D molecular model complexes, respectively. Using computational design techniques, we then explored occupation of these positions by combinations of all 20 possible natural amino acids to obtain a short list of the most favourable amino acid substitutions at each position. Other residue types, identified from position-dependent frequency analyses of variation in a multiple sequence alignment of lysC homologues were then included in the candidate list. By limiting the number of permitted mutations in this way at each of the nine positions, the theoretical maximum size of the library could be reduced to 2,160 combinations, which were then constructed experimentally. A total of 6,720 colonies were isolated to ensure adequate screening coverage of the library. The crude extracts were appropriately diluted and incubated for 40 min in reaction medium containing malate as the sole substrate available to the recombinant LysC mutants. Positive hits identified after a first screening round were then tested again using the same procedure as further confirmation of their activity on malate. A total of 14 clones, corresponding to 2% of the total number of mutants screened, were found to express kinase mutants able to catalyse the phosphoryl transfer reaction from ATP to malate at rates of between 2 to 6.8 μmol.min^-1^ per mL of crude extract. Mutant sequencing eliminated five redundant sequences among the positive hits, yielding a total of nine unique LysC mutants that were active on malate (Fig 4). Sequence analysis also revealed systematic mutation of the glutamate residue at position 119. This glutamate residue is known to be highly conserved within the aspartate kinase family. In LysC, the E119 side-chain carboxylate engages in a salt bridge interaction with the α–NH3^+^ group of the cognate substrate aspartate.[9,24,25] Replacement of E119 by a serine residue was observed in eight of the nine unique malate kinase sequences. An E434V mutation that was introduced fortuitously was also identified in three of the sequences [6]. Purified pET28 plasmids harbouring genes coding for the best mutant LysC V115A:E119S:E434V and the wild-type LysC were used to transform fresh *E*. *coli* cells. Proteins were overexpressed and purified for further characterisation.

### Origins of the improved catalytic performance of the best mutant

The best mutant LysC V115A:E119S:E434V showed an 86 fold increase in catalytic efficiency towards (L)-malate ((*k*_cat_/*K*_m_)_malate_) as compared to that on (L)-aspartate ((*k*_cat_/*K*_m_)_aspartate_).[6] The contributions of side-chain replacements at the three residue positions in the wild-type enzyme to the increase in catalytic efficiency on (L)-malate of the LysC V115A:E119S:E434V mutant were explored by a comparison of kinetic parameters with those of E119 mono-mutants [6] and those of re-constituted V115A:E119S, V115A:E119C and E119S:E434V double mutants (Table 1). The effect of ATP concentration on the kinetics was also studied (Table 1). In comparison with the wild-type enzyme working on its natural aspartate substrate, *K*_m_ and substrate inhibition *K*_i_ values for ATP are not significantly different in any of the mutants investigated (S1 Fig).

**Table 1:** Kinetic parameters on (L)-malate and (L)-aspartate for LysC wild-type and mutants.

Enzyme	ATP		L-malate			
	*K*_m_ (mM)	*K*_i_ (mM)	*k*_cat_ (s^-1^)	K_m_ (mM)	*k*_*cat*_*/K*_*m*_ (mM^-1^ s^-1^)	Mutant versus wild-type (*k*_*cat*_*/K*_*m*_)_mal_ / (*k*_*cat*_*/K*_*m*_)_asp_
E119 (wild-type)^[a]^	nd	nd	0	nd	nd	0
E119Q^[a]^	nd	nd	0.03± 0.004	38.5± 0.71	0.001	0.06 x 10^−2^
V115A E119S	0.41 ± 0.03	8.3 ± 0.50	4.96 ± 0.07	9.5 ± 0.41	0.52	31 x 10^−2^
V115A E119C	0.35 ± 0.01	8.07 ± 0.27	5.5 ± 0.16	10.29 ± 1.02	0.53	32 x 10^−2^
E119S E434V	nd	nd	1.59 ± 0.35	13.11 ± 1.88	0.12	7 x 10^−2^
V115A E119S E434V^[a]^	0.67 ± 0.02	8.59± 0.29	6.82 ± 0.14	8.31 ± 0.52	0.82	49 x 10^−2^
	**ATP**		**L-aspartate**			
	*K*_m_ (mM)	*K*_i_ (mM)	*k*_cat_ (s^-1^)	*K*_m_ (mM)	*k*_*cat*_*/K*_*m*_ (mM^-1^ s^-1^)	Mutant versus wild-type (*k*_*cat*_*/K*_*m*_)_asp_ / (*k*_*cat*_*/K*_*m*_)_asp_
E119 (wild-type) ^[b]^	0.33	21	13.2± 0.4	7.9± 0.24	1.7	
V115A E119S E434V^[a]^			1.14 ± 0.2	119.5 ± 29.8	0.010± 0.003	5.6 x 10^−3^

^[a]^ (L)-malate kinetic parameter data from Table S5.2 of Walter et al. [6]

^[b]^ (L)-aspartate kinetic parameter data from Table S5.2 of Walter et al. [6]

nd: not determined

As LysC had no detectable catalytic activity on (L)-malate, values for the basal turnover number and Michaelis constant used to calculate binding energy gain were determined from the kinetic data obtained experimentally for the E119Q mutant on (L)-malate.[6] Taking the E119Q mutant as a convenient replacement for the wild-type LysC reference, the additional binding energy gained through (V115A:Q119S:E434V) triple mutation that is used to stabilise the (L)-malate substrate in the malate kinase enzyme reaction transition state can be approximated from *k*_cat_ and *K*_m_ parameter ratios for the two enzymes to be -2.3 kcal mol^-1^ (Table 2). This is slightly higher than a calculated binding free energy value of -1.7 kcal mol^-1^ at 298K for (L)-malate when acting as an inhibitor of wild-type LysC with a thermodynamic equilibrium dissociation constant (*K*_i_) of 53 mM.[9]

**Table 2:** Utilisation of additional (L)-malate binding energy gained by LysC mutation in stabilising enzyme-substrate and enzyme transition-state complexes.

Reference Enzyme	Enzyme Mutant	ΔΔ*G*_*S*_ ^[a]^ [kcal mol^-1^]	$\Delta \Delta G_{T}^{‡}\;$^[b]^ [kcal mol^-1^]
E119Q	E119G	-0.61	-1.85
E119Q	E119A	-0.77	-1.86
E119Q	E119S	-0.65	-1.88
E119Q	E119C	-0.41	-2.54
E119Q	V115A:E119S	-0.83	-2.20
E119Q	V115A:E119C	-0.78	-2.30
E119Q	E119S:E434V	-0.64	-1.71
E119Q	V115A:E119S:E434V	-0.91	-2.30
E119S	V115A:E119S	-0.17	-0.32
E119S	V115A:E119S:E434V	-0.25	-0.43
E119S	E119S:E434V	+0.02	+0.17
E119S	E119C	+0.24	-0.66
E119S	V115A:E119C	-0.13	-0.45
V115A:E119S	V115A:E119S:E434V	-0.08	-0.11

^[a]^ ΔΔ*G*_*S*_ is the difference in the enzyme-substrate (ground-state) stabilisation energies of the mutant and reference (mutant) complexes.

^[b]^ $\Delta \Delta G_{T}^{‡}\;$ is the corresponding change in the enzyme-substrate transition-state complex stabilisation energy similarly with respect to the free enzyme and substrate species.

On the assumption of quasi-equilibrium substrate binding, ΔΔ*G*_*S*_ is given by *RT* ln(*Km*′/*Km*), where *Km*′ and *Km* are the Michaelis constants of the mutant and reference enzymes for (L)-malate, respectively.$\;\Delta \Delta G_{T}^{‡}\;=\;-\Delta \Delta G^{‡}-\Delta \Delta G_{S},\;$ where ΔΔ*G*^‡^ is the change in enzyme reaction activation energy. From transition-state theory, ΔΔ*G*^‡^ can be calculated as $RT\text{ln}\_\left(k_{cat\;}^{\prime }{/k}_{cat}\right)$, where $k_{cat}^{\prime }$ and *k*_*cat*_ are the respective maximum turnover numbers of the mutant and reference enzymes. *R* is the ideal gas constant. The temperature (*T*) was taken as 298 K. Kinetic parameters for (L)-malate are listed in Table 1.

**Fig 5:**
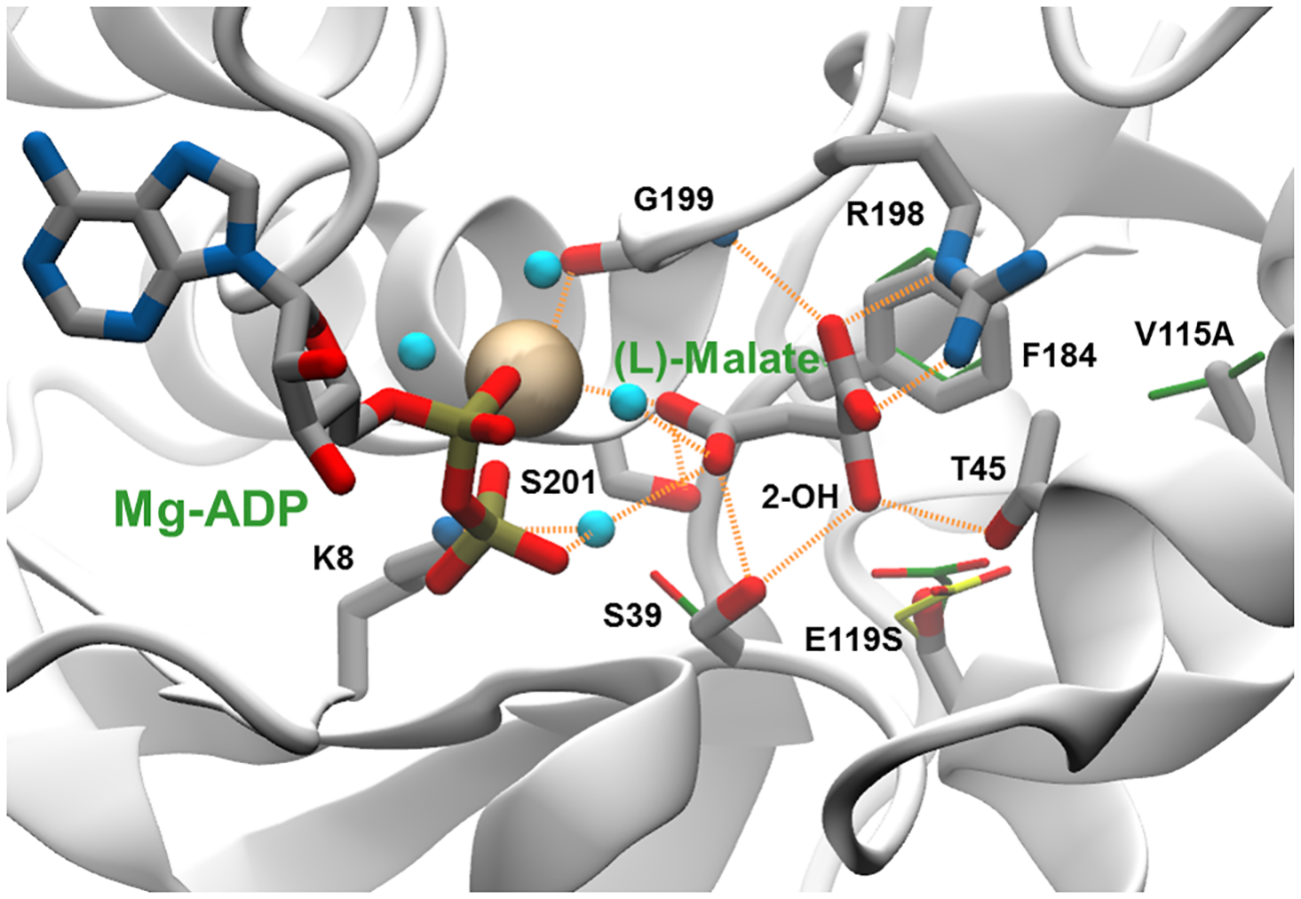
Enzyme binding site interactions in a modelled complex of the LysC E119S:V115A double mutant with (L)-malate and Mg-ADP. A network of direct and water-mediated interactions between (L)-malate and enzyme residues and Mg-ADP is depicted as dashed line orange vectors connecting donor and acceptor heavy-atom co-ordinate positions. The Mg^2+^ ion is shown as an ochre-coloured space filling sphere, and water molecules mediating substrate binding and metal ion co-ordination interactions as cyan-coloured spheres. Atoms in (thick) stick representations of (L)-malate, ADP and labelled mutant enzyme side-chains are coloured according to element type: carbon, grey; nitrogen, blue; oxygen, red; and phosphorus, orange. The oxygen atom of the 2-OH hydroxyl group of (L)-malate that replaces the charged α-NH3 group in (L)-aspartate is indicated. Carbon atoms in (thin) stick side-chains representations in an overlay of the X-ray structure of the R-state *holo* complex of the wild-type enzyme with (L)-aspartate and Mg-ADP (PDB code 2j0w) are shown in green. For comparison an alternative E119 side-chain conformation observed in the inactive T-state *apo* form of the wild type enzyme (PDB code 2j0x), re-constructed on the 2j0w backbone, is depicted in (thin) stick representation with yellow-coloured carbon atoms.

An increase of up to -1.9 kcal mol^-1^ in additional binding energy available for transition-state stabilisation, when compared to the E119Q mutant reference, was found for E119G, E119A and E119S mutants, and -2.5 kcal mol^-1^ for the E119C mutant (Table 2). Energy gains used in the stabilisation of the (L)-malate substrate ground state were in the range -0.4 to -0.8 kcal mol^-1^ for these mono-mutants, but lower than that (-0.9 kcal mol^-1^) for the V115A:Q119S:E434V triple mutant (Table 2). The side-chain conformation of Glu119 present at a central position in a 24-residue helix is notably distorted in the salt-bridge interaction with the (L)-aspartate substrate α-amino group in the R-state of the ternary complex with the Mg-ADP reaction co-product.[24] The χ1 dihedral angle value is close to zero (-8°), compared to a value of -75° in the crystallographic structure of LysC in the *apo* T-state (PDB code 2j0x).[24] The shift in Glu119 sidechain orientation, brought about through the adoption of an energetically less favourable rotomeric conformer induced by binding of the cognate (L)-aspartate substrate, is overlain on a modelled complex of the LysC V115A:E119S double mutant with (L)-malate in Fig 5. Similar distortion of an uncharged glutamine sidechain at position 119 engaged in an analogous interaction with uncharged (L)-malate 2-hydroxyl group might be expected to be less likely in the absence of additional substrate binding energy from a charge-charge interaction. The presence of shorter less sterically cumbersome side-chains in E119G, E119A, E119S and E119C mutants may explain their improved catalytic properties towards (L)-malate compared to the E119Q mutant.

The exchange of Val115 by alanine in the double mutants V115A:E119S and V115A:E119C respectively increased the malate kinase catalytic efficiency (*k*_cat_/*K*_m_) by a factor of 3.1 and 2.3 compared to the corresponding single mutants (Table 1). This improvement was now inversely brought about by a notable decrease in the *K*_m_ for (L)-malate in the V115A:E119C mutant, and a more pronounced increase in *k*_cat_ in the V115A:E119S variant. As a result, the V115A:E119C and V115A:E119S double mutants exhibited nearly identical *k*_cat_/*K*_m_ values of approximately 0.5 mM^-1^ s^-1^ on (L)-malate, equivalent to 30% of the catalytic efficiency of the wild-type enzyme when acting on (L)-aspartate.

**Fig 6:**
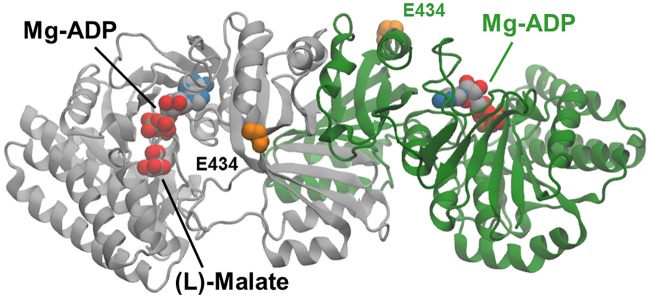
Molecular model of a ternary complex of the V115A, E119S Lys-C mutant with (L)-malate and Mg-ADP. Cartoon representations of the dimer subunits are coloured grey and green. (L)-malate and Mg- ADP are shown as van der Waals spheres coloured according to element type: carbon, grey; nitrogen, blue; oxygen, red; phosphorus, orange; magnesium, ochre Side-chain atoms in the E434 residues at the enzyme surface are highlighted as orange-coloured van der Waals spheres. E434 lies approximately 27Å from the (L)-malate substrate molecule bound in one of the two active sites.

As can be seen in Fig 5, residue position 115 lies behind Phe184, and no direct contact is made with (L)-malate. The V115A mutation may act so as to increase the effective volume available for movement of the Phe184 aromatic side-chain, thereby permitting improved binding of the (L)-malate substrate. The separation distance of 4.6 Å between the Ser119 Oγ side-chain atom and the (L)-malate 2-OH hydroxyl is too long to provide for hydrogen bonding. However, rotation of the Ser39 side-chain and interaction of the Thr45 side-chain can allow the formation of two hydrogen bonds from these residue side-chains with the 2-OH group illustrated in Fig 5. The combined effect of these small movements aided by the removal of the potentially sterically obstructive Glu119 side-chain is to create a more compact binding site in which catalytically productive binding of the (L)-malate substrate can occur. Introduction of the E434V mutation in the V115A:E119S double mutant led to a further increase in *k*_cat_/*K*_m_ towards (L)-malate. Glu434 lies on the enzyme surface, approximately 26 to 30 Å distant from the active site (Fig 6). The results of electrostatics calculations on the V115A:E119S and V115A:E119S:E434V mutants containing bound (L)-malate, summarised in Table 3, demonstrate a favourable relative increase in the total electrostatic energy of interaction for (L)-malate of approximately -0.08 kcal mol^-1^. This is in accord with the estimated additional stabilisation of the substrate ground-state (-0.08 kcal mol^-1^) calculated directly from the experimentally determined mutant Michaelis constants for (L)-malate (Table 2). The additional improvement in catalytic efficiency brought about by the E434V surface mutation is consistent with a favourable long range electrostatic effect on catalysis. However, the E119S:E434V double mutant exhibited slightly reduced activity compared to the E119S single mutant, and the beneficial effect of the E434V mutation on catalysis appears to be dependent upon the presence of the V115A mutation.

**Table 3:** Calculated (L)-malate electrostatic binding interaction energies in the V115A:E119S and V115A:E119S:E434V Ec-LysC mutant ternary complexes with Mg-ADP.

Mutant	Δ*E*_*Coulomb*_	Δ*G*_*Polar*_	Δ*G*_*Elec*_	ΔΔ*G*_*Elec*_	*ε*
V115A: E119S	+108.63	-112.00	-3.37		4
V115A: E119S:E434V	+99.28	-102.73	-3.45	-0.08	4
V115A:E119S	+217.25	-221.66	-4.41		2
V115A:E119S:E434V	+198.56	-203.04	-4.48	-0.07	2

Total electrostatic (L)-malate binding interaction energies (Δ*G*_*Elec*_) and their Coulombic (Δ*E*_*Coulomb*_) and polar solvation (Δ*G*_*Polar*_) difference energy components in units of *kcal mol*^-1^ are calculated for different values of the dielectric constant for the protein interior, *ε*.

## Conclusions

Many methods have been developed to screen kinase activity based on the detection of either phosphorylated products, ATP consumption, or ADP formation. Most of these methods involve specific, often difficult to handle and expensive materials, such as radiolabelled ATP, fluorescently-labelled peptides, tailored antibodies and/or specialised laboratory equipment such as for example a fluorescence/luminescence plate reader, flow cytometer or NMR spectrometer.[11,26–28] A miniaturised end-point ADP-based colorimetric assay has been recently reported.[29] This method involves the addition of a recombinant mouse nucleotidase to the reaction medium in order to selectively release the β-phosphate of the ADP generated by the kinase reaction. The amount of released inorganic phosphate, which is correlated with kinase activity, is then detected using malachite green. In comparison with our procedure, this method suffers from the requirement for the removal of any pre-existing free phosphate present in the enzyme extract following cell disruption. Furthermore, this method has not to date been tested in the screening of a large library of variants. The screening assay developed here is operationally relatively uncomplicated, and was found to be sufficiently reproducible and sensitive for kinase activity screening applications. The method was used to screen a library of more than 6,000 variants, and to isolate a triple mutant of an aspartate kinase possessing a novel (L)-malate kinase activity. The catalytic constant (*k*_cat_/*K*_m_) of the triple mutant enzyme on malate was only 2 fold lower than that of the wild-type enzyme acting on its natural substrate, (L)-aspartate [6]. More remarkably, this protein variant was almost ineffective on L-aspartate. Kinetic analysis of re-constituted component single and double mutant enzymes allowed the contributions of individual mutation sites to the overall catalytic performance to be delineated. Although the procedure described here was adapted to kinase engineering, it is expected to be of more general application in the engineering of any type of ATP-utilising enzyme.

## Supporting information

S1 Table List of oligonucleotides used in this study. (DOCX)

S1 Fig Estimation of Michaelis (K_m_) and inhibition (K_i_) constants for ATP in wild-type lysC and mutant enzymes from double-reciprocal Lineweaver-Burk plots. (DOCX)
