# Supplementary material for: A generic HTS assay for kinase screening: Validation for the isolation of an engineered malate kinase
Source: PLoS One. 2018 Feb 20;13(2):e0193036. doi: 10.1371/journal.pone.0193036 (PMC5819781; doi:10.1371/journal.pone.0193036)
Supplement: S1 Table — (DOCX) [file pone.0193036.s001.docx]

# Supplementary Information

**A Generic HTS Assay for Kinase Screening: Validation for the Isolation of an Engineered Malate Kinase**

Romain Irague^1,2^, Christopher M. Topham^1,2^, Nelly Martineau^1,2^, Audrey Baylac^1,2^, Clément Auriol^1,2^, Thomas Walther^1,2^, Jean-Marie François^1,2^, Isabelle André^1,2^*, Magali Remaud-Siméon ^1,2^*

^1^ Laboratoire d’Ingénierie des Systèmes Biologiques et Procédés, LISBP, Université de Toulouse, CNRS, INRA, INSA, Toulouse, France. 135, avenue de Rangueil, F-31077 Toulouse cedex 04, France

^2^ Toulouse White Biotechnology. Parc technologique du canal, Bâtiment NAPA CENTER B, 3, rue des Satellites, F-31400 Toulouse, France

***** Corresponding authors

E-mails : [isabelle.andre@insa-toulouse.fr](mailto:isabelle.andre@insa-toulouse.fr);[remaud@insa-toulouse.fr](mailto:remaud@insa-toulouse.fr)

**S1 Table. List of oligonucleotides used in this study.**

| pETseq_for | atgcgtccggcgtaga |
| --- | --- |
| pETseq_rev | gctagttattgctcagcgg |
| lysC_A40A-T45S | gtgcgtttagttgtcctctcggcttctgctgggatctctaatctgctggtcgctttagctgaa |
| lysC_A40S-T45T | gtgcgtttagttgtcctctcgagttctgctgggatcactaatctgctggtcgctttagctgaa |
| lysC_A40S-T45S | gtgcgtttagttgtcctctcgagttctgctgggatctctaatctgctggtcgctttagctgaa |
| lysC_V115S-E119D | ctgacagatgagctgagcagccatggcgatctgatgtcgaccctg |
| lysC_V115S-E119G | ctgacagatgagctgagcagccatggcgggctgatgtcgaccctg |
| lysC_V115S-E119N | ctgacagatgagctgagcagccatggcaatctgatgtcgaccctg |
| lysC_V115S-E119Q | ctgacagatgagctgagcagccatggccagctgatgtcgaccctg |
| lysC_V115S-E119S | ctgacagatgagctgagcagccatggctcgctgatgtcgaccctg |
| lysC_V115A-E119D | ctgacagatgagctggccagccatggcgatctgatgtcgaccctg |
| lysC_V115A-E119G | ctgacagatgagctggccagccatggcggcctgatgtcgaccctg |
| lysC_V115A-E119N | ctgacagatgagctggccagccatggcaatctgatgtcgaccctg |
| lysC_V115A-E119Q | ctgacagatgagctggccagccatggccagctgatgtcgaccctg |
| lysC_V115A-E119S | ctgacagatgagctggccagccatggctcgctgatgtcgaccctg |
| lysC_V115V-E119D | ctgacagatgagctggtcagccatggcgatctgatgtcgaccctg |
| lysC_V115V-E119G | ctgacagatgagctggtcagccatggcgggctgatgtcgaccctg |
| lysC_V115V-E119N | ctgacagatgagctggtcagccatggcaatctgatgtcgaccctg |
| lysC_V115V-E119Q | ctgacagatgagctggtcagccatggccagctgatgtcgaccctg |
| lysC_V115V-E119S | ctgacagatgagctggtcagccatggctcgctgatgtcgaccctg |
| lysC_F184G | gcttagttataacccagggaggtatcggtagcgaaaataaag |
| lysC_T195R | aaaataaaggtcgtacaacgaggcttggccgcgg |
| lysC_T195S | aaaataaaggtcgtacaacgtcgcttggccgcgg |
| lysC_S201A | cttggccgtggaggcgccgattatacggcagc |
| lysC_T359D | cttgataccacaggttcagactccactggcgatacgtt |
| lysC_T359V | cttgataccacaggttcagtctccactggcgatacgtt |
| Ec_lysC_clon_for | cacgaggtacatatgtctgaaattgttgtctcc |
| Ec_lysC_clon_rev | cttccaggggatccagtatttactcaaac |
