# Supplementary material for: A generic HTS assay for kinase screening: Validation for the isolation of an engineered malate kinase
Source: PLoS One. 2018 Feb 20;13(2):e0193036. doi: 10.1371/journal.pone.0193036 (PMC5819781; doi:10.1371/journal.pone.0193036)
Supplement: S1 Fig — (DOCX) [file pone.0193036.s002.docx]

# Supplementary Information

**A Generic HTS Assay for Kinase Screening: Validation for the Isolation of an Engineered Malate Kinase**

Romain Irague^1,2^, Christopher M. Topham^1,2^, Nelly Martineau^1,2^, Audrey Baylac^1,2^, Clément Auriol^1,2^, Thomas Walther^1,2^, Jean-Marie François^1,2^, Isabelle André^1,2^*, Magali Remaud-Siméon ^1,2^*

^1^ Laboratoire d’Ingénierie des Systèmes Biologiques et Procédés, LISBP, Université de Toulouse, CNRS, INRA, INSA, Toulouse, France. 135, avenue de Rangueil, F-31077 Toulouse cedex 04, France

^2^ Toulouse White Biotechnology. Parc technologique du canal, Bâtiment NAPA CENTER B, 3, rue des Satellites, F-31400 Toulouse, France

***** Corresponding authors

E-mails : [isabelle.andre@insa-toulouse.fr](mailto:isabelle.andre@insa-toulouse.fr);[remaud@insa-toulouse.fr](mailto:remaud@insa-toulouse.fr)

**S1 Figure: Estimation of Michaelis (K_m_) and inhibition (K_i_) constants for ATP in wild-type lysC and mutant enzymes from double-reciprocal Lineweaver-Burk plots**

**
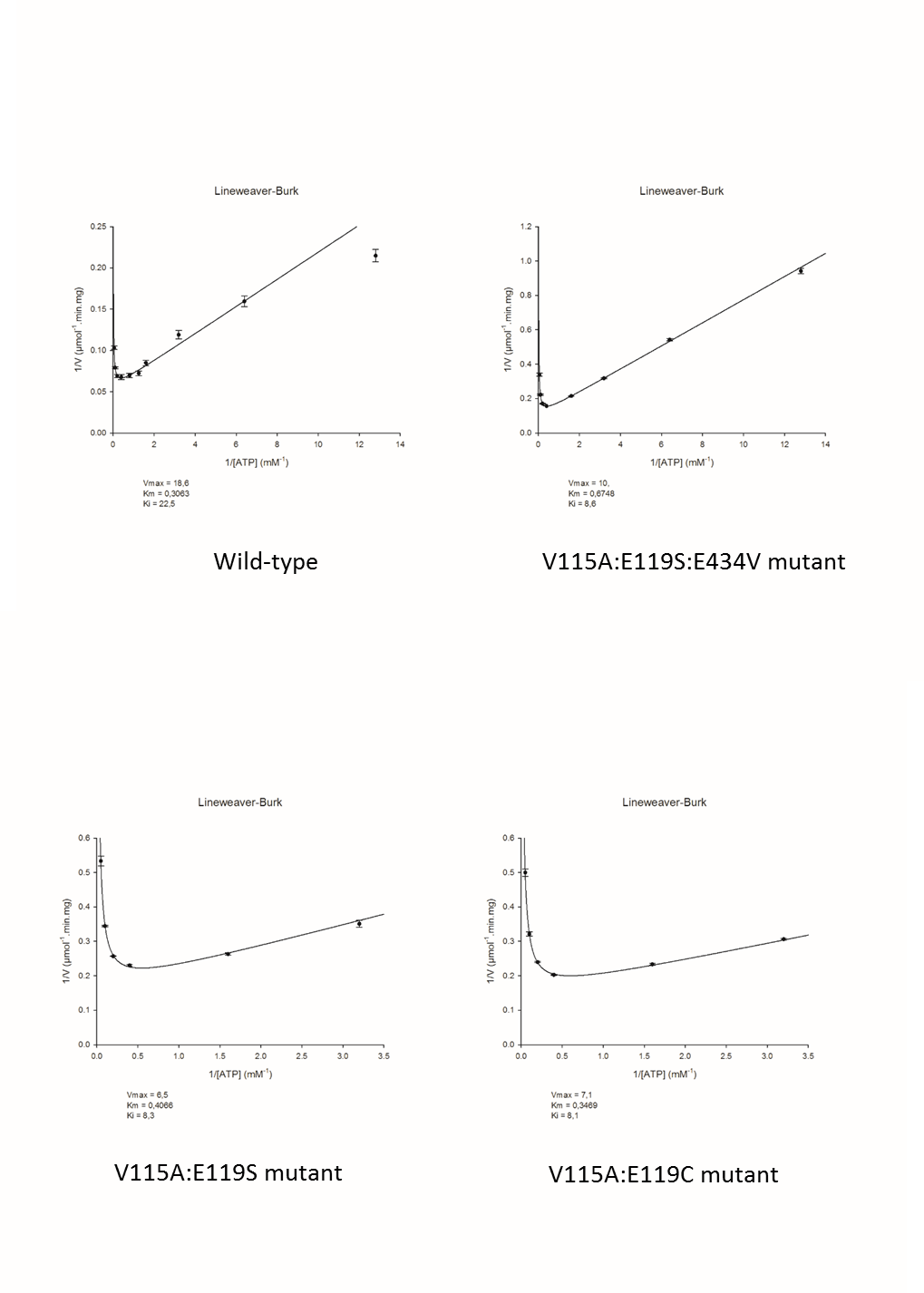
**
